# Food-Borne Outbreak Investigation and Molecular Typing: High Diversity of *Staphylococcus aureus* Strains and Importance of Toxin Detection

**DOI:** 10.3390/toxins9120407

**Published:** 2017-12-20

**Authors:** Sarah Denayer, Laurence Delbrassinne, Yacine Nia, Nadine Botteldoorn

**Affiliations:** 1 Scientific Service of Food borne Pathogens, Scientific Institute of Public Health (WIV-ISP), 1050 Brussels, Belgium; Laurence.Delbrassinne@wiv-isp.be (L.D.); Nadine.Botteldoorn@wiv-isp.be (N.B.); 2 Laboratory for Food Safety, Anses, Université Paris-Est, 94701 Maisons-Alfort, France; yacine.NIA@anses.fr

**Keywords:** *Staphylococcus aureus enterotoxins*, SEA, SEB, SEC, SED, staphylococcal food poisoning (SFP), pulsed-field gel electrophoresis (PFGE)

## Abstract

*Staphylococcus aureus* is an important aetiological agent of food intoxications in the European Union as it can cause gastro-enteritis through the production of various staphylococcal enterotoxins (SEs) in foods. Reported enterotoxin dose levels causing food-borne illness are scarce and varying. Three food poisoning outbreaks due to enterotoxin-producing *S. aureus* strains which occurred in 2013 in Belgium are described. The outbreaks occurred in an elderly home, at a barbecue event and in a kindergarten and involved 28, 18, and six cases, respectively. Various food leftovers contained coagulase positive staphylococci (CPS). Low levels of staphylococcal enterotoxins ranging between 0.015 ng/g and 0.019 ng/g for enterotoxin A (SEA), and corresponding to 0.132 ng/g for SEC were quantified in the food leftovers for two of the reported outbreaks. Molecular typing of human and food isolates using pulsed-field gel electrophoresis (PFGE) and enterotoxin gene typing, confirmed the link between patients and the suspected foodstuffs. This also demonstrated the high diversity of CPS isolates both in the cases and in healthy persons carrying enterotoxin genes encoding emetic SEs for which no detection methods currently exist. For one outbreak, the investigation pointed out to the food handler who transmitted the outbreak strain to the food. Tools to improve staphylococcal food poisoning (SFP) investigations are presented.

## 1. Introduction

Staphylococcal food poisoning (SFP) is one of the most common reported food-borne diseases and results from the consumption of food in which coagulase positive staphylococci (CPS), mostly *Staphylococcus aureus*, have grown and produced enterotoxins (SEs).

*Staphylococcus aureus* strains can produce a range of different exotoxins, among them the enterotoxins, which causes vomiting with or without diarrhea and are responsible for SFP. The enterotoxins, together with toxic shock syndrome toxin-1 (TSST-1), also function as superantigens (SAgs). By binding to both the Vβ chain of T cell receptors and to MHC class II molecules on antigen presenting cells these activate a broad range of T cells in an antigen nonspecific manner, resulting in massive cytokine secretion and subsequent systemic inflammation and toxic shock syndrome (TSS) which can lead to a fatal outcome [1,2].

The genes encoding the different enterotoxins are carried and disseminated by different mobile genetic elements, i.e., prophages, plasmids, pathogenicity islands (SaPIs), enterotoxin gene clusters (egc) and the staphylococcal cassette chromosome (SCC) [2,3]. To date, 23 distinct SEs have been described based on their antigenicity and these are referred to as SEA to SElY in the chronological order of their discovery [2,4]. Only a subset of SEs possesses emetic activities [3,4,5]. Among the 23 SEs identified and reported in literature, only five SEs (SEA, SEB, SEC, SED and SEE) have been well characterized, and are detectable using commercially available assays or in-house developed methods [6,7]. Recent work showed that other SEs (SEG, SEH, SEI, SER, SES and SET) have been identified as potential agents of food poisoning as well [4,5]. Besides the emetic SEs, multiple SE like toxins (SEls) have been reported, which lack the ability to cause emesis or for which the emetic potential remains to be tested [8]. Therefore, their role in SFP remains unclear. Because of their stability at high temperatures (crude enterotoxin A, 100 °C for 2 h; in mushrooms, 28 min at 121 °C) SEs are not completely destroyed during food processing (15 s at 72 °C) and they are considered to be a potential biological threat [2,9]. In addition, SEs are resistant to many environmental conditions (low pH, freezing, drying) in which CPS strains do not survive and they are resistant to human proteolytic enzymes, retaining their activity in the digestive tract after ingestion [2]. In previous SFPs, these toxins have been mainly qualitatively reported. Unfortunately, quantitative data in terms of type and concentration of SEs in food matrices involved in SFPs are scarce, with the lowest suspected dose of SEA reported being about 0.36–0.66 ng/mL in chocolate milk [10]. Other reports suggest that as less as 20–100 ng of enterotoxin A may cause symptoms in a susceptible adult [2]. Recent investigations using dose-response modeling of staphylococcal enterotoxins using outbreak data revealed that 6.1 ng of SEA is sufficient to induce effects in 10% of an exposed population (benchmark dose (BMD10)) [11].

Staphylococcal food poisoning is characterized by a rapid onset of symptoms that can occur within 30 min after ingestion of SE-contaminated foods [12]. The typical incubation period is 2 to 7 h, with symptoms resolving in about 12 h. Nausea, stomach cramps, vomiting and sometimes diarrhea are the typical SFP symptoms, while fever is seldom reported. For sensitive persons belonging to the YOPI (young, old, pregnant, immunosuppressed) group, in particular, hospitalization might be necessary. Symptoms depend on the total amount of toxin ingested, on the overall health condition of the affected person but also on the toxin type [2].

Diagnosis of SFP is mainly based on the enumeration of >10^5^ *S. aureus* cfu/g and/or the detection of SEs in food leftovers, and on the isolation of identical *S. aureus* clones from both patients and food leftovers [13]. Typically, SFP is associated with improper handling of cooked or processed foods, rather than raw foods, followed by storage under conditions which allows the growth of *S. aureus* and production of enterotoxins [3]. Foods that favor growth of CPS are characterized by high protein content. These include meat and meat products, poultry and egg products, milk and dairy products, salads, bakery products -particularly cream-filled pastries and cakes-, and sandwich fillings and all have been frequently incriminated in SFP outbreaks [2,14,15,16,17,18]. On the other hand, staphylococcal enterotoxin production is influenced by various factors including pH, water activity, temperature and other parameters [8].

Food handlers carrying enterotoxin producing-*S. aureus* in their noses or on their hands are regarded as the main source of food contamination via manual contact or through respiratory secretions. In fact, *S. aureus* is a common commensal bacterium of the skin and mucosal membranes of humans with estimates of 20% and 30% persistence, respectively [19]. However, *S. aureus* can also enter the dairy chain as it is also a likely contaminant of milk when dairy cattle, sheep and goats are affected by mastitis [2,20]. These multiple origins of CPS reflect the need for straightforward epidemiological analysis in SFP investigation and source identification.

Different molecular typing methods have been used to compare *Staphylococcus aureus* strains in outbreak investigations, including MLVA, spa-typing and pulsed-field gel electrophoresis (PFGE) [21]. Until now, PFGE remains the Gold Standard for typing of CPS although new molecular techniques in outbreak investigation and strain typing such as next generation sequencing (NGS) become more popular and seem promising for future surveillance [22,23,24,25]. Currently, PFGE is less expensive as compared to NGS and does not require exhaustive bio-informaticians or software. However, NGS and newly developed software tools to analyze whole genome sequence data might allow rapid detection of toxin genes and determination of MLST-, Spa-, MLVA- and maybe even pulsotypes.

Over years 2011–2015, data reported by the European Food Safety Authority and the European Centre for Disease Control (EFSA and ECDC) showed that bacterial toxins are the third most important causative agent group in Europe. In 2011, 2012, 2013, 2014 and 2015, bacterial toxins represented 12.9%, 14.5%, 16.1%, 16.1% and 19.5% of the reported food-borne outbreaks in the European Union [14,15,16,17,18]. Over these 5 years, Staphylococcal enterotoxins (SEs) were involved in half of the reported bacterial toxins outbreaks, whilst the remaining involved the emetic toxin of *Bacillus cereus* (*B. cereus*) or enterotoxins produced by *Clostridium perfringens* or *B. cereus*. In 2015, 16 EU Member States reported 434 SFP, representing 51% of food-borne outbreaks caused by bacterial toxins and 9.9% of all outbreaks [18].

Here we report on three food poisoning outbreaks due to staphylococcal enterotoxins which occurred in 2013 in Belgium. Staphylococcal enterotoxins were characterized and quantified in the food leftovers in order to determine the toxin dose causing disease. The obtained quantitative data could help risk managers to set food safety criteria for SEs in food products different from those currently available in the legislation, and thus prevent SFP. Molecular typing of human and food isolates was used to assess the link between patients and the consumption of a suspected foodstuff and to investigate food handler involvement.

## 2. Results

### 2.1. Epidemiological Investigation

In 2013, three outbreaks were reported to the National Reference Laboratory for Food-borne outbreaks in Belgium (NRL FBO), for which CPS and SEs were identified as the causative agent of the outbreak based on both human and food sample results.

- The first outbreak (outbreak A), took place in an elderly home that housed 111 residents aged between 68 and 99 years. In total 28 residents (24 female and four male), started vomiting within three hours after lunch. Vomiting was followed by diarrhea. Their lunch consisted of meat loaf, cod and mashed potatoes. The mashed potatoes were prepared in the morning and stored for two hours in a water bath at 90 °C until lunch.
- The second outbreak (outbreak B) occurred after a barbecue meal where 18 participants suffered from vomiting and diarrhea upon 6 hours after consumption of various foods. The catering service responsible for the food delivered a similar barbecue meal at a second barbecue event, but no cases were reported there. Interestingly, a current failure of the refrigerating device occurred, but temperature was verified and claimed to be conform by the caterer. None of the food handlers were ill before, during or after the event.
- In the third outbreak (outbreak C) six out of seven children aged between nine months and two years started vomiting within 1 h after eating a mashed carrot-potato mixture with fish at a kinder garten. One child who consumed only one spoon of the meal did not become ill. All children were sent to the hospital and two of them were in shock. All recovered fast and left the hospital the same day. The mashed carrot-potato mixture consisted of leftovers from the lunch of the previous day, which was cooled at room temperature before storage in the fridge. The fish was stored frozen and heated (steamed) just before consumption.

### 2.2. Laboratory Investigation

Since vomiting symptoms started rapidly (i.e., less than 6 h) upon consumption of the implicated foodstuffs, laboratory investigations focused on emetic toxin producing-food pathogens, namely *B. cereus* and CPS.

#### 2.2.1. Bacterial Investigation of Human Samples

None of the human cases or food handler’s samples collected from the three outbreaks were positive for emetic toxin producing *B. cereus*. CPS was isolated in all three outbreaks as extensively presented in the Table 1.

In both outbreaks A and C involving elderly people and children, respectively, CPS was isolated from stool from human cases and from nasal/throat swabs from food handlers (Table 1). In outbreak B, involving participants to a barbecue meal, none of the human case or food handler samples contained CPS. In outbreak A, enterotoxigenic CPS harboring the *sea*, *sed*, *seg*, *sei*, *sej* and *ser* genes (Table 1) were isolated. In addition, 10 CPS strains were isolated from 7 food handlers that prepared the meal for the residents, three -belonging to two food handlers (FH4 and FH7)- of which were non-enterotoxigenic. Two of the food handlers carried CPS isolates harboring the *sea* and *seh* genes (Table 1, FH1 and FH3). A fifth employee (FH2) carried a CPS strain that harbored *sec*, *seg* and *sei* enterotoxin genes. From the two remaining food handlers (FH5 and FH6), CPS strains harboring *seg*, *seh* and *sei* genes and, *seg* and *sei* genes, respectively were isolated. In outbreak C, except for the vomit, enterotoxigenic CPS could be isolated from stools of all human cases and food handler’s (i.e., nanny) samples. These all harbored the *sea*, *seg* and *sei* genes. The CPS strains isolated from both nasal and throat swabs from the nanny possessed identical properties.

#### 2.2.2. Bacterial Investigation of Food Samples

None of the food or water samples tested were positive for emetic toxin producing *B. cereus*. CPS were detected in various food leftovers for each of the outbreaks (Table 1). For outbreak A, only one out of eighteen food samples resulted positive for CPS (270 cfu/g). For outbreak B, 5 out of 15 food samples contained CPS at various levels ranging from 10^2^ to 7.2 × 10^6^ cfu/g (Table 1). For outbreak C, one out of two food samples resulted positive for CPS with levels higher than 1.5 × 10^7^ cfu/g found in mashed potatoes with carrots (Table 1). Two CPS strains did not possess any of the *se*-genes tested, whereas the remaining isolates harbored one up to six *se*-genes as presented in Table 1.

#### 2.2.3. Toxin Investigation (SE Detection, Identification and Quantification)

The results for the detection, identification and quantification of SEs in food samples are presented in Table 1. For outbreak A, SEA was quantified at a low concentration of 0.019 ng/g, in the mashed potatoes that contained CPS (Table 1). For outbreak B, SE could only be detected in leftovers from sausages and the potato mixture. Both SEA and SEC were quantified in the potato mixture at levels up to 0.015 ng/g and 0.132 ng/g, respectively. For the sausages, toxin levels were probably below the detection limit. Unfortunately, no food sample was left for SE detection in the case of outbreak C. The use of SET-RPLA on VIDAS SET2 positive samples only detected SEC in the potato mixture for outbreak B, otherwise resulted negative.

#### 2.2.4. Comparison of CPS Strains from Human and Food Origin

**Figure 1:**
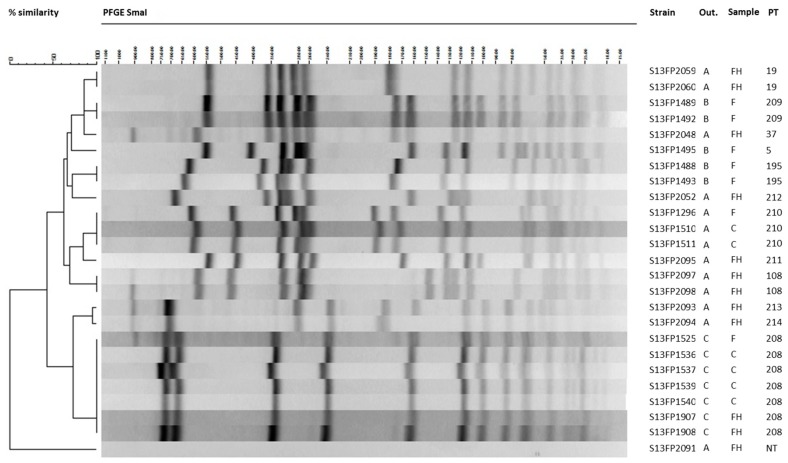
Comparison of food and human isolates from outbreak A, B and C using pulsed-field gel electrophoresis (PFGE). Legend: Food (F); Human Case (C); Food Handler (FH); Outbreak (Out.); Pulsotype (PT).

All *se*-positive CPS isolates from the three outbreaks were subjected to pulsed-field gel electrophoresis (PFGE) for further comparison (Figure 1).

Many different pulsotypes were circulating in the human cases, food handlers and foods involved in the three outbreaks. In outbreak A, many different pulsotypes (i.e., 19, 37, 211, 212, 213) were circulating within the food handlers but none was identical to the pulsotype 210 which was found for both the food and human isolates from cases (Figure 1, Table 1). In outbreak B, the five food isolates belonged to three pulsotypes (i.e., 5, 195, 209). For outbreak C, all the strains isolated from the mashed potatoes, human cases and the food handler had an identical pulsotype (i.e., 208).

## 3. Discussion

Here, we describe three confirmed food poisoning outbreaks due to CPS. All can be considered as outbreaks with strong microbiological and epidemiological evidence according to the European Food Safety Authority (EFSA) nomenclature [26]. Microbiological analysis excluded the implication of emetic *B. cereus* as a causative agent.

The most common SE responsible for SFP worldwide is SEA [3]. This was also the case for the three reported outbreaks with findings on enterotoxin A on both phenotypic and genotypic level among the CPS strains from human cases and food leftovers. Potato-based food products, including mashed potatoes and a potato mixture intended to be eaten cold, were the incriminated foods in the three outbreaks. Staphylococcal enterotoxins could be quantified in these food products, except for outbreak C where the analysis could not be performed due to an insufficient amount of leftovers. For outbreak B, SEA was accompanied by higher levels of SEC. The combined presence of SEA and SEC has been reported earlier in SFP, but to our knowledge not in potato-based foods [21]. On the other hand, potato-based products have been involved in previous SFP outbreaks but they involved other SEs such as SEA and SEH [21,27].

The amount of enterotoxin that causes illness is not yet specifically known. Assuming a portion size of 100 g, levels ranging from 1.9 ng (mashed potatoes outbreak A) to 14.7 ng (potatoes outbreak B) were quantified and resulted in vomiting and subsequently diarrhea. These levels are far below those reported previously where as little as 20–100 ng of enterotoxin have been reported to be effective in causing SFP [2,28]. Depending on the V-beta loops present on T cells, and the V-beta profiles induced upon binding of an SE, different superantigenic activities and thus SAg-related diseases are observed. This variability in human response makes it difficult to determine the SE dose causing symptoms in an individual person [1,3,29]. For outbreak A, the presence of multiple enterotoxins, that might have synergistic effects, cannot be excluded since other enterotoxin genes (*sea*, *sed*, *seg*, *sei*, *sej* and *ser*) were detected in the *S. aureus* isolates. Although some of these, i.e., SEG, SEI and SER, possess emetic activities and have a potential role in SFP, methods for the detection of these toxins in food still need to be developed [4,5]. For outbreak C, where it was not possible to determine toxin presence directly in the food leftovers due to an insufficient amount of sample, the high levels of CPS detected in the potato-carrot mixture (>10^7^ cfu/g) suggest that very high concentrations of toxin type A were present. Since this outbreak implied toddlers aged between 9 months and 2 years, and SEs are known to induce SAg-mediated TSS, the outcome of this SFP could have been much more severe, especially taking into account the high variability in sensitivity of individuals [1,2,3,29].

Different analytical tools have been developed for the detection and quantification of SEs, including immunological and mass spectrometry methods, each of which has its limitations especially on availabilities of specific antibodies, peptides and standards [8,13,30,31]. At this time, commercially available ELISA methods allow routine detection of only five classical SEs, SEA to SEE, in food matrices, with and without identification of the toxin types SEA to SED [6,7,8]. For outbreak A, no SEs were detected in the food using the commercially available VIDAS SET2 method although SEA toxin was detected using Ridascreen^®^ SET and an in-house ELISA method developed by the EU-RL for Staphylococci. This can be explained by the low amount of toxin present (0.019 ng/g), being under the limit of detection of the VIDAS SET2 method which is set at 0.25–1 ng/g [28]. The negative results obtained using SET-RPLA are in agreement with previous observations made by Nia and co-workers [7], describing less sensitivity as compared to VIDAS SET2 and Ridascreen.

The availability of reliable, rapid and sensitive SE detection methods is important in SFP investigations but also to generate data that allow risk managers to set food safety criteria for SEs and thus prevent SFP. For outbreak A, SEA was detected in mashed potatoes containing CPS as less as 270 cfu/g of food, which is lower than the levels (10^5^ cfu/g) allowed in Regulation 2073/2005 for cheese, milk or whey powder (food hygiene criterion) [32]. Currently, no European criteria exist for other food products. This confirms previous observations that pre-formed SEs still can resist in heat-treated food products, whereas *S. aureus* may be eliminated and thus enumeration of CPS only cannot be used to characterize a SFP outbreak [13]. For outbreak B, *sea*-positive CPS were present at levels ranging from 10^2^–10^3^ cfu/g in various foods besides the highly contaminated potato mixture, but no SEA was detected. A transfer of SEA-producing CPS strains through cross-contamination during storage at the setting cannot be excluded, or the level of SEA in these foods was below the detection limit of all methods used. Highly sensitive SE detection methods also allow to get new insights in the pathogenesis of *S. aureus* and food safety control measures that need to mitigate risks of staphylococcal intoxications. The detection of both emerging and established toxins under different conditions, including strain based differences, even at levels below those causing food poisoning symptoms, could elucidate their potential role in SFP [33,34]. Perreira and co-workers described the existence of low-enterotoxin-producing staphylococci that could still produce sufficient amounts of enterotoxins in food to cause SFP, which demonstrates the interest of the low detection limits of a method [35]. Strain dependent ability of CPS to produce different levels of enterotoxins, but also the influence of the matrix on the produced SE levels has been demonstrated for SED and SER [33]. Recent work shows that new detection methods even allow the detection of biologically active SED in foods [36]. Sensitive SE detection methods would also allow to study the risks following chronic exposures of individuals to low levels of toxins and translate the knowledge on the effect of relevant amounts of toxins in concrete prevention and intervention measures in the food chain [37]. Although the presence of an enterotoxin gene is not a valid indication of SE protein expression, PCR could be a valuable screening tool for staphylococcal isolates and food matrices, and could give an indication whether there is a probability of enterotoxin presence in a food product even for newly described SE genes for which no SE detection method exists to date. Typically, SFP is associated with improper handling of cooked or processed foods, followed by storage in conditions allowing growth of *S. aureus* and production of enterotoxins. The presence of enterotoxin genes in CPS isolates from healthy carriers, including food handlers, highlight the possible risk of food product contamination during food preparation and processing. Moreover, many of our isolates possessed enterotoxin genes different from the classical SEs, of which the role in food intoxications is not always known. Indeed, PCR allowed the detection of the genes *sea*, *sec* or *sed* encoding the classical enterotoxins in the food and human isolates while genes encoding for other SEs (*seg*, *seh*, *sei*, *sej*, *ser* and *sep*) were also detected. For some of the latter (*seh*, *sep* and *ser*), an increase in prevalence in CPS isolates from clinical cases, healthy carriers, including food handlers has been reported, indicating their importance in SFP [38,39]. In outbreak A, three food handlers resulted positive for *S. aureus* isolates carrying the *seh* gene, which has been described in previous outbreaks in Japan and Norway demonstrating the need for methods that are able to detect SEH directly in foods [8,27,38,39]. For the outbreaks described here, none of the CPS strains isolated from food carried the *seh* gene. The combination of *se* genes, further referred to as *se*-gene profile, can also give a first indication of an existing link between a contaminated food and a human case. This is shown in outbreaks A and C for which the same *se*-gene profile, *sea-sed-seg-sei-sej-ser* and *sea-seg-sei* respectively, was observed between the food isolate and one or more human CPS isolates. Nasal, throat or stool isolates from individual patients or food handlers showed identical *se*- gene profiles.

The use of molecular typing methods such as PFGE is important since presence of SEs or *se* gene carriage might lack discriminatory power to link a contaminated food to a patient, food handler or food business operator. For instance, two isolates (one nasal, one throat) from a food handler in outbreak A showed identical *se*-gene profiles, but the strains belonged to different but closely related pulsotypes 213 and 214. This was also the case for isolates from two different food handlers (FH1 and FH3) showing identical *se*-gene profiles (*sea*, *seh*) but belonging to pulsotypes 37 and 19, respectively. On the other hand, PFGE confirmed that the enterotoxigenic *S. aureus* strains (*sea*, *seg*, *sei*) isolated from the human cases in outbreak C were identical to the food isolate, all belonging to pulsotype 208. Moreover, the strain isolated from the food handler, in this case the nanny, showed the same pulsotype meaning that the nanny probably transferred the strain into the mashed potatoes. Although PFGE profiles (i.e., 210) linked the consumed food to the human cases for outbreak A, it remains unknown how the strain got into the food for outbreaks A and B. During the SFP investigation, only one isolate per patient, food handler or food sample was selected which could explain why no link could be established. Enterotoxigenic *S. aureus* may constitute a resident member of the intestinal microbial community and colonized persons can harbor multiple *S. aureus* strains in the microflora for a longer period of time, which was demonstrated in isolates from children [40]. Other studies demonstrate that 7–30% of individuals colonized with *S. aureus* possess multiple strain types within one body niche and from one up to six different genotypes were identified per individual [41]. Similarly, Acco and co-workers demonstrated multiple *S. aureus* carriage for food handlers in the food industry by testing 3 isolates per food handler [42]. This means that food products are possibly contaminated with several isolates. Therefore, it is recommended to include multiple isolates per sample in SFP investigations. It has to be noted that comparison of genomes not only can demonstrate involvement of a food handler but importantly can also serve to exclude it.

## 4. Conclusions

Three SFP outbreaks due to enterotoxin type A-producing *S. aureus* were described, where levels of SEA from 1.9 ng to 14.7 ng in potatoes induced symptoms. Investigations included the analysis of nasal or throat samples from people handling the food, as well as samples from food components or food leftovers. In depth molecular typing demonstrated a high diversity of circulating CPS strains which carry *se*-genes coding for enterotoxins different from the conventional toxins SEA to SEE. Since several CPS strains might be carried by human hosts and thus be potentially transferred to food products, source tracking in SFP investigation using molecular typing (e.g., PFGE) should include multiple isolates per sample. Since SEs not only have their role in SFP, but are also a possible threat to both food safety and food security if they are produced in a purified form that can be used for intentional contamination, it is crucial to develop reliable, sensitive and rapid methods for the detection of SEs different from SEA–SEE. This will also be important to establish food safety criteria for the prevention of SFP and to increase the number of reports on strong evidence SFP. In the meantime, PCR methods give complementary information during SFP investigation. Food-borne outbreak investigations require a well-coordinated approach, within a multidisciplinary team, gathering together people from animal and human health but also epidemiologists and food microbiologists.

## 5. Materials and Methods

### 5.1. Outbreak Investigation and Sampling

In Belgium, inspectors of the Federal Agency for Safety of the Food Chain (FASFC) are responsible for food investigations and sampling of foods, whereas medical doctors of the Belgian Communities are responsible for collecting human samples. Four outbreaks occurred in 2013 of which three have been described here. A case was defined as anybody who had lunch at the location where the outbreak took place and who experienced vomiting episodes within 1–6 h:

- Outbreak A: reported to the Health Inspection Service by medical doctors, occurred in an elderly home.
- Outbreak B: reported to the inspector of the FASFC by the head of the catering company, occurred in a temporary mass gathering-barbecue.
- Outbreak C: reported to the Health Inspection Service by medical doctors, occurred in a kindergarten.

In outbreaks A, B and C, vomiting was observed 3, 6 and 1 h after food consumption, respectively. Epidemiological information such as age and symptoms, but also the timeline and circumstances of the outbreaks, were gathered from patients (outbreaks A and B), parents of patients (outbreak C), the director of the elderly home (outbreak A) or food handlers (outbreak A, B and C) using a standard questionnaire. All collected information was transmitted to the Belgian National Reference Laboratory of Food-borne Outbreaks (NRL-FBO).

Microbiological analyses were performed on food and human samples at the NRL-FBO. Leftovers from a variety of food items eaten by the residents (outbreak A), participants of the barbecue meal (outbreak B) or children at the kindergarten (outbreak C) were collected.

- For outbreak A, 18 different composed dishes or food components similar to those consumed the day of the outbreak (mashed potatoes, meat loaf, two samples of cod, cooked liver pasta) or the day before the outbreak (vanilla pudding, cheese, rice, bread, potatoes, mashed potatoes, horse filet, sausages, 3 sauces, mixed chicken with broccolis and leek) were sampled.
- For outbreak B, 15 food items similar to those consumed by the victims were sampled, some of which were stored frozen until sampling. These included meat products (bovine steak, sausages, lamb meat), poultry (chicken chops), vegetables (potatoes, mixture of carrots/cucumber/white cabbage), bakery products (bavarois), fish products (two different salmon preparations, sardines), crustacean (scampi) and mousse based on salmon, ham, duck or crab. For this outbreak, 2 ground water samples were also collected.
- For outbreak C, only one sample containing a small quantity of leftovers consisting in a mashed carrot-potato mixture with frozen fish was collected.

In addition, stool samples (A = 9, B = 5, C = 4) from affected persons or from food handlers (B = 4) and vomit specimen (A = 1) were collected for the three outbreaks. Finally, in the course of the investigation of outbreaks A and B, food handlers were also screened for *S. aureus* colonization, resulting in 16 (A = 15, C = 1) nasal and 16 (A = 15, C = 1) throat swab samples.

### 5.2. Laboratory Investigation

#### 5.2.1. Enumeration of Coagulase Positive Staphylococci and *B. cereus* in Food and Water Samples

The enumeration of coagulase positive *Staphylococcus aureus* (CPS) was performed on a test portion of 25 ± 1 g according to ISO 6888-1:1999 [43]. Since only 2 g of carrot-potato mixture was sampled for outbreak C, 18 mL of buffered peptone water was added to obtain the initial suspension for the enumeration (1:10 dilution). The enumeration of *B. cereus* was performed similarly according to ISO 7932:2004 [44].

One hundred milliliters of ground water (outbreak B) was filtered using a 0.45 µM MilliPore. The filter was deposited on Baird Parker Agar (Bio-Rad, Marnes-la-Coquette, France) and incubation and confirmation of coagulase by means of rabbit plasma of suspect isolates was performed as described in ISO 6888-1:1999 [44].

#### 5.2.2. Detection and Quantification of Staphylococcal Enterotoxins in Food Samples

All food leftovers from outbreak A were subjected to toxin detection, while for outbreak B this was only done on food leftovers that resulted positive for CPS. The low quantity of food sample from outbreak C did not allow a toxin detection. Extraction of enterotoxins was conducted on 25 g test portions as described in the European Screening Method (Version 5) of the European Union Reference Laboratory for CPS including *S. aureus* (EURL CPS, Anses, Paris, France) and SEA to SEE detection was conducted by use of VIDAS^®^ SET 2 kit (bioMérieux, Marcy l’Étoile, France) according to the manufacturer’s instructions [45]. All samples positive for toxin detection using VIDAS SET 2 were subsequently tested for the SEs A, B, C, and D using reversed passive latex agglutination (SET-RPLA Toxin Detection kit, Oxoid nv, Groot-Bijgaarden, Belgium) according to the manufacturer’s instructions with a single modification in that Brain Heart Infusion broth (BHI, Sigma-Aldrich, Overijse, Belgium) was used instead of Tryptone Soy broth.

All foodstuffs, except for one for which no sample was left (potato-carrot mixture, outbreak C), that resulted positive for CPS have been subjected for confirmation of SEs by Ridascreen^®^ SET A, B, C, D, E (R-Biopharm, Darmstadt, Germany) and a specific ELISA. Briefly, quantification and identification of SEs types (SEA, SEB, SEC, SED) was performed using a quantitative indirect sandwich-type ELISA. Briefly, double sandwich ELISA types were used for SEA, SEC, and SED whereas a single sandwich type was used for SEB. Specific commercially available antibodies (Toxin Technology, Sarasota, FL, USA) were used as coating (references SLAI101, SLBI 202, SLCI 111, SLDI 303) and probing antibodies (references LAI101, LBC 202, LCI 111 and LDI 303). The presence of enterotoxins was revealed by immunoglobulins coupled to horseradish peroxidase (goat-anti rabbit antibodies coupled to peroxidase) and determined by a colorimetric measurement at 405/630 nm after addition of the substrate, ABTS—H_2_O_2_ (KPL). Quantification was performed using SEs standards purchased from Toxin Technology, Sarasota, Florida, USA (batch 120794A for SEA, 61499B1 for SEB, 113094C2 for SEC and 12802D for SED). Finally, internal positive and negative controls were prepared from milk extract and analyzed in the same manner as that of the samples.

#### 5.2.3. Isolation of Coagulase Positive *S. aureus* and *B. cereus* from Human Samples

A loopful of each sample of human stool or vomit specimen was streaked directly on Mannitol Egg Yolk Polymyxin Agar (MYP agar, Bio-Rad, Marnes-la-Coquette, France) and Baird-Parker agar (Bio-Rad, Marnes-la-Coquette, France) in order to search for *B. cereus* and CPS, respectively. Nasal and throat swabs were directly cultivated on Baird-Parker agar for the isolation of CPS. Suspect CPS isolates were confirmed for the production of coagulase by means of rabbit plasma as described in ISO 6888-1:1999 and confirmation of Bacillus cereus was performed as described in ISO 7932:2004 [43,44].

#### 5.2.4. Genotypic and Phenotypic Characterization of Coagulase Positive *Staphylococcus aureus*

One CPS isolate per sample (patient, food handler, or food product) was included in the investigations.

The production of staphylococcal enterotoxins A, B, C and D was characterized for food and human isolates using the SET-RPLA method (SET-RPLA Toxin Detection kit, Oxoid nv, Groot-Bijgaarden, Belgium) as described by the manufacturers’ instructions. For each sample that tested positive for CPS, five individual colonies were characterized.

All food and human isolates (1 for each sample) were further characterized by Polymerase Chain Reaction (PCR). Genomic DNA was prepared using the InstaGene kit (Bio-Rad, Marnes-la-Coquette, France) according to the manufacturer’s recommendations.

Isolates were confirmed as *S. aureus* using a 23 S rDNA specific PCR assay and analysed for the presence of 11 SE genes (*sea*, *seb*, *sec*, *sed*, *see*, *seg*, *seh*, *sei*, *sej*, *sep* and *ser*) using two conventional multiplex PCR assays, as described previously [21].

To compare food and human isolates, a pulsed-field gel electrophoresis (PFGE) was conducted on se-positive CPS isolates, using SmaI (Life Technologies, Ghent, Belgium) as restriction enzyme, as previously described [21].

## Figures and Tables

**Table 1:** Overview of CPS counts in food samples, detection of CPS in human samples and typing results of isolates during the investigations of outbreaks A, B and C.

Outbreak	Origin (F, C, FH) *	Matrix	CPS Investigation (cfu/g, D, ND)	SE Detection ^a^		SE Quantification (ng/g) ^b^	*se*-Genes	Pulsotype
				Strain Isolate (SE-type, ND)	Food (D, ND)			
Outbreak A	F	Mashed potatoes	270	SEA, SED	D	SEA 0.019	*sea*, *sed*, *seg*, *sei*, *sej*, *ser*	210
	F	Diverse (17 samples)	ND		ND		*No strain isolated*	
	C	Stool 1	D	SEA			*sea*, *sed*, *seg*, *sei*, *sej*, *ser*	210
	C	Stool 2	D	SEA			*sea*, *sed*, *seg*, *sei*, *sej*, *ser*	210
	FH ^1^	Swab throat	D	SEA			*sea*, *seh*	37
	FH ^2^	Swab throat	D	SEC			*sec*, *seg*, *sei*	212
	FH ^3^	Swab Nose/Throat	D	SEA			*sea*, *seh*	19
	FH ^4^	Swab Nose	D	ND			*negative for the tested se-genes*	N/A
	FH ^5^	Swab Nose	D	ND			*seg*, *seh*, *sei*	213
	FH ^5^	Swab throat	D	ND			*seg*, *seh*, *sei*	214
	FH ^6^	Swab Nose	D	ND			*seg*, *sei*	211
	FH ^7^	Swab Nose/Throat	D	ND			*negative for the tested se-genes*	N/A
	20 FH/7 C	Swab/Stool	ND				*No strain isolated*	
Outbreak B	F	Chicken	1700	SEA, SEC	ND	N/A	*sea*, *sec*	195
	F	Sausage	1300	SEA	D	N/A	*sea*	209
	F	Bovine meat	900	ND	ND	N/A	*sea*	209
	F	Potato preparation	7,200,000	SEA, SEC	D	SEA 0.015, SEC 0.132	*sea*, *sec*	195
	F	Dessert (pie)	100	ND	ND	N/A	*sep*	5
	F	Diverse (11 samples)	ND		N/A		*No strain isolated*	
	4 FH/5 C	Stool (9 samples)	ND				*No strain isolated*	
Outbreak C	F	Mashed potatoes with carrots	>15,000,000	SEA	N/A		*sea*, *seg*, *sei*	208
	F	fish	ND		N/A		*No strain isolated*	
	C	Stool 1	D	SEA			*sea*, *seg*, *sei*	208
	C	Stool 2	D	SEA			*sea*, *seg*, *sei*	208
	C	Stool 3	D	SEA			*sea*, *seg*, *sei*	208
	C	Stool 4	D	SEA			*sea*, *seg*, *sei*	208
	FH	Swab Nose/Throat	D	SEA			*sea*, *seg*, *sei*	208
	C	Vomit	ND				*No strain isolated*	

F: Food; C: Human Case; FH: Food Handler; N/A: Not Analysed; D: Detected; ND: Not Detected with the detection limit being 100 cfu/g in food; SE type: Staphylococcal Enterotoxin type. *: Human samples originating from a same C or FH are marked with identical numbers in superscript. ^a^ commercial method (VIDAS and/or Ridascreen on food, SET-RPLA on strain); ^b^ in house ELISA method (LOD SEA 0.0015 ng/g, LOD SEB 0.011 ng/g, LOD SEC 0.002 ng/g, LOD SED 0.0088 ng/g).
